# Changes in Atlantic climatic regulation mechanisms that underlie mesozooplankton biomass loss in the northern Baltic Sea

**DOI:** 10.1016/j.heliyon.2024.e31268

**Published:** 2024-05-14

**Authors:** Heta Rousi, Julia Fält-Nardmann, Pekka Niemelä, Jari Hänninen

**Affiliations:** a Archipelago Research Institute, Biodiversity Unit, FI-20014, University of Turku, Finland; b Kevo Subarctic Research Institute, Biodiversity Unit, FI-20014, University of Turku, Finland; c Institute of Forest Zoology, Dresden University of Technology, Pienner Straße 7, D-01737, Finland; d Institute of Dentistry, University of Turku, Lemminkäisenkatu 2, FI-20520, Turku, Finland

**Keywords:** Copepods, Cladocerans, Rotifers, Functional groups, North atlantic oscillation, Regime shift

## Abstract

The effects of climate-induced, long-term changes on mesozooplankton biomasses were studied based on monitoring data collected since 1966 in the northern Baltic Sea. We found that the biomasses of marine and brackish mesozooplankton had decreased significantly from 1966 to 2019, and a remarkable biomass and functional biodiversity loss took place in the mesozooplankton community. Our results put emphasis on the impact of two climate-driven regime shifts for the region's mesozooplankton community. The regime shifts took place in 1975 and 1976 and in 1989 and 1990, and they were the most important factor behind the abrupt biomass changes for marine mesozooplankton and total and marine Copepoda. Only the latter regime shift influenced the biomasses of brackish Copepoda, marine Cladocera, and total Rotifera. The decreasing length of the ice-cover period drove the decrease of the biomass of limnic *Limnocalanus macrurus* (Copepoda), while the winter North Atlantic Oscillation was behind biomass changes in the total and the brackish Cladocera. These findings may have important implications for planktivorous fish, such as Baltic herring, particularly in terms of their impact on commercial fishing.

## Highlights

• We studied the effects of climate on mesozooplankton functional group time series.
• Changes in North Atlantic Oscillation were the ultimate cause of biomass decreases.
• A mesozooplankton functional biodiversity loss, caused by climate change, occurred.

## Graphical abstract

**Figure:**
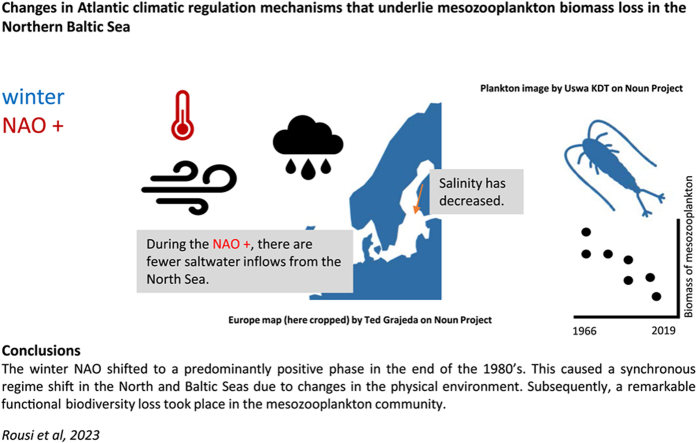

## 1 Introduction

Globally, climate change-induced effects have profoundly affected marine ecosystems [e.g. Refs. [[1], [2], [3]]]. The average estimated global sea surface temperature rise between 1850 and 2020 has been 0.88 °C [4]. Temperature changes have been most pronounced near the polar regions, such as the Baltic Sea [e.g. Ref. [3]]. The Baltic Sea is a semi-enclosed brackish water basin with a considerable north-south gradient of salinity due to the discharge of numerous rivers and limited salt-water intrusions from the North Sea [5]. Climate change has further influenced Baltic Sea salinity conditions through increased precipitation-driven, river-runoff alterations and fewer salt-water impulses from the North Sea [e.g. Refs. [[6], [7], [8]]].

Seawater salinity is the most important controller of Baltic Sea biodiversity, including zooplankton community biomass and abundance, as it affects the osmotic stress in fauna and flora [[9], [10], [11], [12]]. Recently, climatic factors in the Atlantic Ocean have been suggested to control the changes in hydrography, and, ultimately, also in the zooplankton of the Baltic Sea [e.g. Refs. [[13], [14], [15]]]. Selective planktivory has been suggested as an alternative explanation or a contributor to salinity-induced changes in zooplankton [e.g. Refs. [[16], [17], [18]]].

The subject of this study, mesozooplankton, is defined as 200–2000-μm sized zooplankton [19]. In this study, we concentrated on the three most representative taxa of the size class found in the Archipelago Sea on the southwest coast of Finland. These were the phylum Rotifera (i.e., rotifers), the suborder Cladocera (i.e., water fleas or cladocerans), and the subclass Copepoda (i.e., copepods), and these represent the most common species. Zooplankton studies have been conducted in the Finnish coastal waters since the early 20th century [e.g. Refs. [12,20,21]]. However, there is only limited information on the zooplankton community and biomass changes. This is especially true for changes concerning functional traits of the species due to global climate change including climate-induced Regime Shifts (RS) and atmospheric-oceanic circulation pattern changes [e.g., the North Atlantic Oscillation (NAO)]. Acknowledging species-specific functionality, however, is important, as species present different types of environmental preferences in terms of, for example, seawater salinity content and temperature. In addition, organisms with a marine or limnic origin live close to their tolerance limits in the brackish environment of the Baltic Sea. Only a few species are well adapted to live in the low-salinity environment of the northern Baltic Sea [e.g. Refs. [1,12]]. Thus, even small changes in environmental conditions will affect the organisms significantly [1].

NAO describes the pressure relationship between Iceland and the Azores [22,23]. From the end of the 1980s, winter month NAO index values have been predominantly positive [22,23]. This relates to the general increase of winter precipitation and temperatures (low pressure) in the Baltic catchment area, and on the contrary, high pressure in the Mediterranean and Middle East areas [24]. The changes in the climate of northwestern Europe induced a synchronous RS in the North Sea and the Baltic Sea marine ecosystems [7,24]. RS are defined as abrupt changes in the state of an ecosystem caused by climate-induced changes in the physical environment [cf [2,7,25]]. Biological RS involve pronounced modifications in the biota [2]. Thus, they are a transition between alternative stable states in ecosystem structure and function [e.g. Ref. [25], and references therein]. Completion of a shift takes several months to a year [25,26]. In the North and Baltic Seas, RS have been found to impact organisms through climate-driven atmospheric and oceanic changes in salinity and temperature [7,24,27]. In addition, RS are linked to direct anthropogenic causes including overexploitation of target populations and subsequent trophic cascades as well as hypoxia or anoxia that are related to excess eutrophication [e.g. Refs. [18,26,28]].

The purpose of this study was to investigate climate-induced mesozooplankton community structure changes, which were measured as realized biomasses of functional groups in the Archipelago Sea, northern Baltic Sea. The hypothesis (H_1_) is that the effect of large-scale climatic anomalies (i.e., the winter NAO and Baltic Sea RS) or phenomena (i.e., the decrease of length of ice cover period), which have occurred in Fennoscandia, are reflected as abrupt level changes in the biomasses of functional groups. Functional biodiversity is here defined as the diversity of functional traits of the studied organisms [29].

The advantage of focusing on functional groups is that they elucidate an organism's niche in the ecosystem and food chain. Additionally, sample sizes grow as species are not treated separately, and the results are not individualized. The Archipelago Sea study site is ideal for studying climate change-related changes because it is situated in a protected area with only minor habitat fragmentation effects. The Seili mesozooplankton time series is the only comprehensive dataset of its kind available, at least in the larger geographical area studied.

## 2 Materials and methods

### 2.1 The study area

**Fig. 1:**
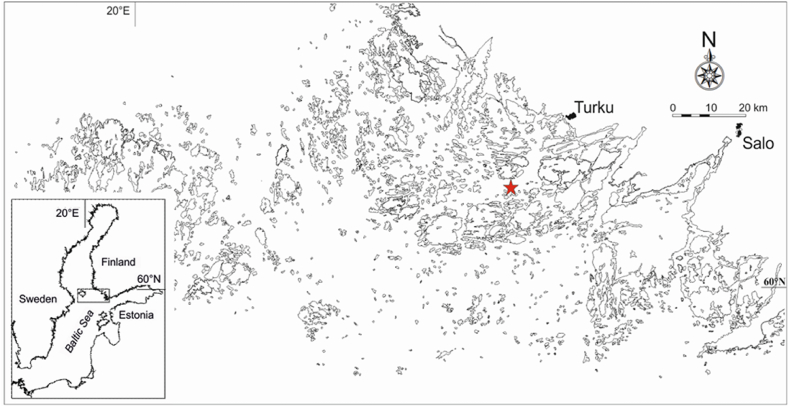
The Baltic Sea and the Archipelago Sea with a location of the Seili zooplankton monitoring site (red star: N60°15′20,39″, E21°57′08,25″) in the middle archipelago. (For interpretation of the references to colour in this figure legend, the reader is referred to the Web version of this article.)

The Baltic Sea is a semi-enclosed brackish water basin [30] with an overall surface area of 415 023 km^2^ (including Kattegat) and a mean depth of 52 m [31]. There is a clear south–northeast salinity gradient in the Baltic Sea varying from 30 PSU in the Danish Straits to only 1 PSU in the Bothnian Bay that affects particularly faunal and floral distributions [e.g. Ref. [32]]. Both saline and freshwater species persist in the Baltic Sea [e.g. Ref. [12]]. The Archipelago Sea is a ragged archipelago at the southwest coast of Finland between the Baltic Proper and the Bothnian Sea (59°45′-60°45′N and 21°00′-23°00′E) in the northern Baltic Sea (Fig. 1).

Depending on the definition of an island, the area consists of up to 60 000 islands [33]. In this respect, it is the biggest archipelago in the world with a complex and a variable topography with mainly wind-driven, water-mass-movement patterns. The total area of the sea is 9436 km^2^ with a water volume of 213 km^3^ and salinity content of 4–6 PSU [34]. The total catchment area of the Archipelago Sea is approximately 8900 km^2^ with a lake area of less than 2 % and arable land of 28 % [35]. The average water depth is only 23 m, as the deepest hollows reach 140 m. The wind-driven sea level variation is generally low with a mostly ±0.5 m variation compared to the theoretical mean level with an insignificant tidal fluctuation [34]. The seasonality is distinguishable to the Archipelago Sea, as in winters, the occurrence of a probability of a 1- to 5-month ice cover is 90 %, but in summers, the seawater temperature can reach 20 °C [5].

### 2.2 Long-term sampling

The Seili research station started its zooplankton monitoring program in 1966. Since then, vertical zooplankton samples have been gathered from the Seili ODAS monitoring station at a depth of 50 m monthly throughout the year, except in some years when samples were not taken during scattered months (Fig. 1, Supplement 1).

During 1966 to 1984, the collection method was a Hensen net (mesh size = 150 μm, mouth diameter = 70 cm), but in 1991, for practical reasons, the method was changed to the modern standard KC plankton net (mesh size = 150 μm, mouth diameter = 35 cm). The used plankton nets and evaluation of their efficiencies to catch mesozooplankton species are described in detail by Vuorinen et al. [13]. The sampling procedure was a single haul from the water column from 25 m to the sea surface. The gathered substance of the net was purged to a 250-ml plastic jar and preserved in buffered formalin (4 %). The samples were examined by standard protocols of HELCOM [36] by the previous Finnish Marine Research Institute (FIMR) during 1967–1984 and by the company Zwerver during 1991–2019. Whenever possible, the samples were identified at either the species or genus level. Copepods were identified into copepodite stages, CI–CIII; copepodite stages CIV–CV; and adult stages, CVI and females/males. Cladocerans were identified as juveniles and/or adults, whereas rotifers were identified into adult stages. Of the rotifers, only the genus *Synchaeta*, including the species *S*. *balthica* Ehrenberg, 1834 and *S*. *monopus* Plate, 1889, was acknowledged because of their large size that the net could collect. The counts were converted to wet weights (μg m^−3^_wwt_) by using the average individual body cavities as an estimate of the weight for each taxon according to the method of Hernroth [19].

Seasonality is well represented in the zooplankton time series. The dataset was comprised altogether of 661 quantitative samples (Supplement 1). No zooplankton samples were gathered during 1986–1990 due to temporal closing of the mesozooplankton monitoring program. More detailed information of the sampling and treating procedures in relation to zooplankton communities in the long-term monitoring program are presented by Mäkinen et al. [37].

### 2.3 Mesozooplankton

According to Vuorinen et al. [13], Rajasilta and Vuorinen [38] and Mäkinen et al. [37], the potential dominant mesozooplankton copepods in the surface water layers of the study area belong to the order Calanoida and include *Acartia* spp. (being mainly *A*. *biﬁlosa* Giesbrecht, 1881 and *A*. *longiremis* Lilljeborg, 1853, in deeper water and some *A*. *tonsa* Dana, 1849) and *Eurytemora afﬁnis* Poppe, 1880. *Acartia* spp. and *E*. *affinis* generally dominate the above-thermocline copepod biomass in the Archipelago Sea [38]. Other less common species are *Limnocalanus macrurus* Sars G. O., 1863, found abundantly in the less-saline Bothnian Sea [38]; *Centropages hamatus* Lilljeborg, 1853; *Pseudocalanus acuspes* Giesbrecht, 1881; and *Temora longicornis* Müller, 1785 that favor a higher salinity and are more common in the central and southern Baltic Sea [31,38]. Common cladocerans include the brackish water species *Bosmina longispina maritima* Leydig, 1860, the marine species *Pleopsis polyphemoides* Leuckart, 1859, genus *Podon* (*P*. *leuckartii* Sars G. O., 1862, *P*. *intermedius* Lilljeborg, 1853), *Evadne nordmanni* Loven, 1836, and an invasive freshwater species *Cercopagis pengoi* Ostroumov, 1891, of Ponto-Caspian origin. In the innermost areas of the archipelago, some freshwater species like *Daphnia* spp. (*D*. *cucullata* Sars G. O., 1862, *D*. *christata* Sars G. O., 1862), *Leptodora kindtii* Focke, 1844, *Sida crystallina* Müller O. F., 1776, and *Chydorus sphaericus* Müller O. F., 1776, can also be found. All cladocerans are prominent species in the above-thermocline plankton community and do not show any pronounced daily vertical migration through the thermocline [11,39]. The most abundant rotifer species in the Archipelago Sea belong to the genera *Keratella* (e.g., *K*. *quadrata* Müller O. F., 1786, *K*. *cochlearis* Gosse, 1851, *K*. *cruciformis* Thompson, 1892) and *Synchaeta* (e.g., *S*. *baltica*, *S*. *monopus*, *S*. *curvata* Lie-Pettersen, 1905, *S*. *fennica* Rousselet, 1909), which have a worldwide distribution and are common inhabitants in freshwater environments and found also in brackish and marine habitats [38].

The distinction of mesozooplankton functional groups is based first on taxonomy at the phylum level (e.g., Copepoda, Cladocera, Rotifera). The phylum level categorization also includes information on the size of the organisms in question. Second, the categorization is based on the groups’ affinity to seawater salinity. Additionally, concerning freshwater copepods (*L*. *macrurus*) and marine (*S*. *monopus*) and brackish (*S*. *baltica*) rotifers, the species positioning in the environmental space also includes information on temperature preference. *L*. *macrurus* thrives in colder waters [40] and rotifers in warmer waters [37]. The biomasses of the functional groups were then compared to biomass changes of total and total marine or total brackish mesozooplankton (Table 1). This was done to reveal which functional component in the total biomass change would explain the trends or changes observed in biomass the best.

**Table 1:**
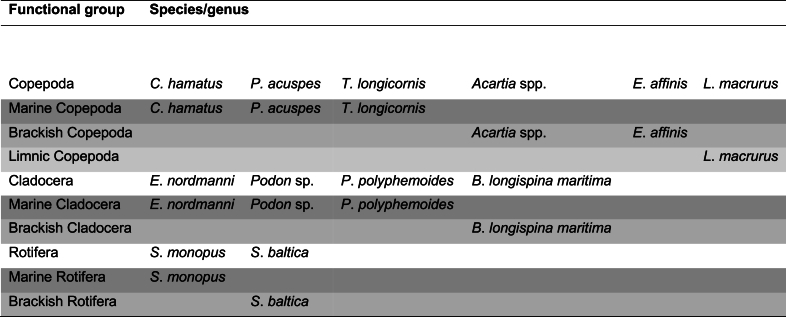
Functional mesozooplankton groups, defined by taxonomy, and seawater salinity and temperature affinities of the species and the species/genera from which they are comprised. Shading from darker to lighter indicates the upper level on which the group belongs: marine mesozooplankton (darkest), brackish mesozooplankton (moderate darkness), and freshwater mesozooplankton (lightest).

The mesozooplankton species or genus comprising the functional groups used in the analyses were the copepods (subclass Copepoda): *Acartia* spp., *C*. *hamatus*, *E*. *affinis*, *L*. *macrurus*, *P*. *acuspes* and *T*. *longicornis*; second, the cladocerans (suborder Cladocera) including: *B*. *longispina maritima*, *Podon* spp., *E*. *nordmanni*, *P*. *polyphemoides*; and third, the rotifers (phylum Rotifera): *S*. *baltica* and *S*. *monopus*. The functional groups formed were a) total, marine and brackish mesozooplankton; b) total copepods, marine copepods, brackish copepods, freshwater copepods; c) total cladocerans, marine cladocerans, brackish cladocerans; and d) total rotifers, marine rotifers, and brackish rotifers (Table 1).

The abundance data concerning actual mesozooplankton also included: a) taxa (e.g., *Keratella* spp.) or developmental stages (e.g., nauplii of copepods), which were smaller than the used plankton net (150 μm), b) rarer taxa (e.g., Bryozoa and *Didinium* sp.), and c) meroplankton (e.g., *Marenzelleria* spp., *Balanus improvisus* Darwin, 1854 and Bivalvia; APPENDIX). These were not converted into biomasses because a) they could not be sampled quantitatively, b) their biomasses were insignificant in proportion to the biomasses of included species, or c) representative meroplankton in the samples, such as Bivalvia larvae and *B*. *improvisus* nauplii, were smaller than the mesh size of the plankton net (APPENDIX).

Additionally, the species observed included two common species that were not included in the analysed functional groups: *D*. *cucullata* and *C*. *pengoi*. *D*. *cucullata* was not included because we could not find a formula for its biomass conversion. *D*. *cucullata* also occurred only in 5.6 % of the samples (APPENDIX). *C*. *pengoi* first appeared in the samples during 1998 and only comprised 0.27 % of the total cladoceran biomass and was calculated according to Antsulevich [41], where the individual conversion factor for *C*. *pengoi* wet weight is 0.3 mg. Due to its relatively insignificant effect on the total cladoceran biomass, *C*. *pengoi* was not included in the analyses. To summarize, in total, 65 taxa were observed from the samples during 1966–2019, but only the 12 most relevant taxa were included. The data remained representative because the included taxa covered the majority of the total mesozooplankton biomass.

### 2.4 Explanatory variables

*Month*, *10-year intervals,* and *RS* were used as class variables in the analyses depending on the model. September was defined, by a default setting in GLIMMIX, as the control month, against which the biomass estimates of other months were compared in the *monthly* iterations.

Regarding the ‘*10-year intervals*’ variable used in the limnic Copepoda and brackish Cladocera analyses, the last interval between 2016 and 2019 was defined as a control period.

In addition, the explanatory random variables used in the models were *Ice days*, averaged winter month (i.e., December, January, February, March) NAO index values or *winter NAO*, and detected *RS* in the Baltic Sea [7]. We considered that the first two variables would describe the best the large-scale atmospheric forcing that the north Atlantic Ocean has directed regionally to the northern Baltic Sea environment in terms of the observed increase in climatic/seawater temperature and to the seawater salinity changes via increased precipitation into the catchment area of the sea [e.g. Refs. [1,15]]. In the case of RS, we regarded that marine ecosystem changes observed both in the North and Baltic Seas [e.g. Refs. [7,8,15]], which also were based on the Atlantic climatic forcing, could have some value in explaining the potential changes in mesozooplankton biomasses of the Archipelago Sea as well.

The number of ice days, starting from December 1970, was extracted from an ice map produced by the Finnish Meteorological Institute from the surroundings of Orhisaari island (N60°16.5′, E21°59.8′) located approximately 4 km north from the mesozooplankton monitoring station in the Airisto inlet. Average winter NAO index values, based on the surface sea-level pressure difference between the Subtropical (Azores) High and the Subpolar (Iceland) Low, were extracted from the calculations of the NOAA (National Oceanic and Atmospheric Association).

(https://www.ncei.noaa.gov/access/monitoring/nao/last accessed October 12, 2021). RS in the Baltic Sea were used as presented in Dippner et al. [7]. RS occurred in 1975/1976 and in 1989/1990, and they were programmed in the data as dummy variables (“0” before 1976, “1” between 1976 and 1989, and “2” from 1990 to 2019).

### 2.5 Statistical analysis

All statistical analyses were performed with GLIMMIX of SAS® 9.4 [42]. GLIMMIX was chosen because, first, it can deal with non-normal data distributions (e.g., log-normal, Poisson or binomial distributions, or any other probability distribution of the ‘*Exponential family*’), which are typical in environmental datasets [43]. Second, GLIMMIX can overcome problems posed by correlated values and non-constant variability, which often are considered as rules in environmental datasets. Third, the linear predictor in GLIMMIX can contain random effects in addition to the usual fixed effects analyses. For a comprehensive presentation of GLIMMIX see Breslow et al. [44].

Before GLIMMIX, we applied SAS Enterprise Guide® 8.3. software [45] to examine the distributions and covariance structures of each variable to ensure that the correct link function and covariance matrix were used in the full analysis. Designated by the outcome, we were able to apply a log-normal distribution as a link function in the analyses. Regarding the biological data, all data points were included in the statistical analyses, and their autocorrelation was assessed. In the original data, the covariance matrix structure proved, without exception, to have a first-order autoregressive structure AR (1), which is typical in bio-environmental time-series data. We used Satterthwaite approximation to define degrees of freedom [42]. The model's fitting was estimated by comparing the changes of values of the *Gener*. *ꭕ*^*2*^/*DF*, *-2 Res* Log *Likehood*, and *AIC* to reach the best parsimony of the model. Models, which had a *General ꭕ*^*2*^/*DF* value > 0 and < 2, −2 *Res* Log *Likelihood* < *Generalized ꭕ*^*2*^, and whose *AIC* value were the smallest in comparison with the alternative models, were regarded as trustworthy [43]. Regarding RS, we conducted custom hypothesis testing with “Estimate” comparisons between the random effects of the distinct RS 0 vs. 1 (1966–1975 vs. 1976–1989), 0 vs. 2 (1966–1975 vs. 1990–2019), and 1 vs. 2 (1976–1989 vs. 1990–2019) and their coefficients.

## 3 Results

**Fig. 2:**
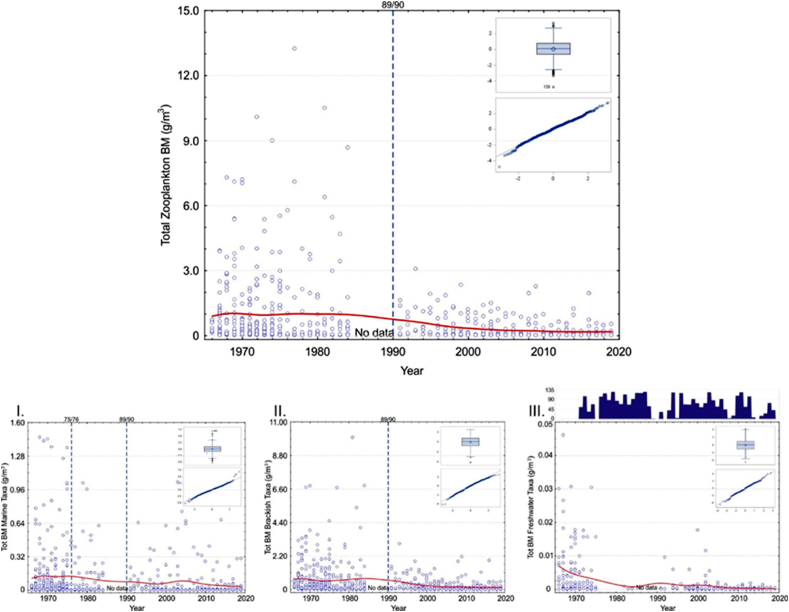
Top panel: Scatterplot for total mesozooplankton biomass change (presented as g m^−3^_wwt_) at the sampling station from 1966 to 2019 (smooth red line is drawn with distance-weighted least squares method). Small panels within the figure express Box plot and Quantile residuals distribution in a viable GLIMMIX model (for a more detailed explanation see text and Table 3, Table 5). If the model did not perform, the residuals were not included. Panels below indicate the same for the main functional groups of mesozooplankton, I. Marine taxa, II. Brackish taxa, and III. Freshwater taxa. Note the different scales on the y-axes. Statistically significant environmental variables in models are presented in each panel. Dashed vertical lines indicate the occasions of confirmed Regime shifts. In panel III, the number of local Ice days are indicated with vertical bars. (For interpretation of the references to colour in this figure legend, the reader is referred to the Web version of this article.)

Brackish mesozooplankton made up some 80–90 % of the total biomass, whereas marine and freshwater mesozooplankton only comprised a minority of the total biomass (APPENDIX). Moreover, in every group, there was an evident continuous biomass decrease with a downward level shift, which seemed to occur between 1984 and 1991 (Fig. 2). Table 2 indicates which environmental variables the functional groups were tested against and the environmental factor's correlation with the mesozooplankton groups.

**Table 2:** Functional mesozooplankton groups, including the upper levels. Additionally, the explanatory factors against which they were tested and response of the tested environmental factors with mesozooplankton groups: + means a positive and **–** means a negative correlation with the explanatory variable. Regime shift (RS) 1 took place in 1975 and 1976 and was caused by a salinity increase following a Major Baltic Inflow. RS 2 took place in 1989 and 1990 and was caused by a shift in the winter NAO into a predominantly positive phase. The duration of sea ice period has shortened since the 1970's. See Table 3, Table 4, Table 5, Table 6, Table 7 for accurate results of the statistical tests.

Functional groups	NAO	RS 1	RS 2	Ice days
Total mesozooplankton			**-**	
Marine mesozooplankton		**+**	**-**	
Brackish mesozooplankton			**-**	
Copepoda		**+**	**-**	
Marine Copepoda		**+**	**-**	
Brackish Copepoda			**-**	
Limnic Copepoda				**+**
Cladocera	**-**			
Marine Cladocera			**-**	
Brackish Cladocera	**-**			
Rotifera			**-**	
Marine Rotifera				
Brackish Rotifera				

**Fig. 3:**
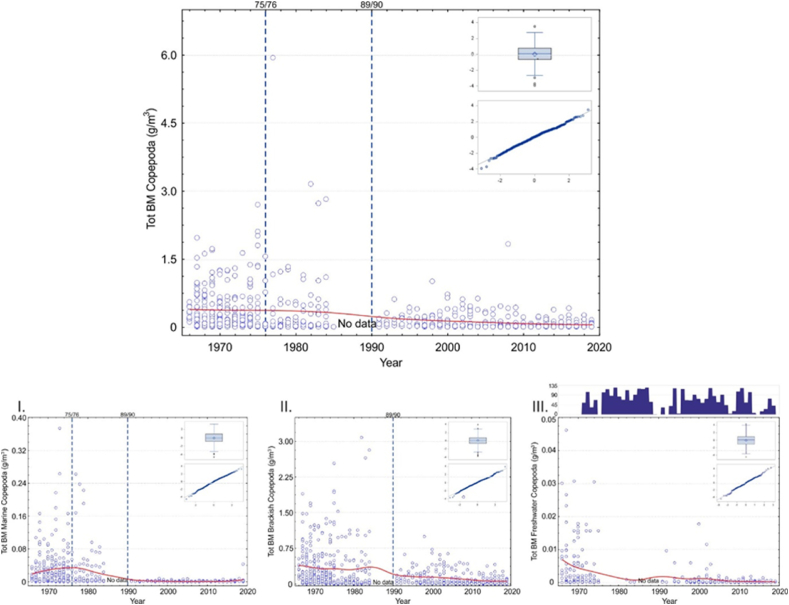
Top panel: Scatterplot for total Copepoda biomass (g m^−3^_wwt_) change at the sampling station in 1966–2019 (smooth red line is drawn with distance-weighted least squares method). Small panels within the figure express Box plot and Quantile residuals distribution of the achieved GLIMMIX model (for a more detailed explanation see text andTable 4, Table 5). Panels below indicate the same for the main functional groups of: I. Marine Copepoda, II. Brackish Copepoda, III. Freshwater Copepoda, and, otherwise, explanations as in Fig. 2. (For interpretation of the references to colour in this figure legend, the reader is referred to the Web version of this article.)

**Fig. 4:**
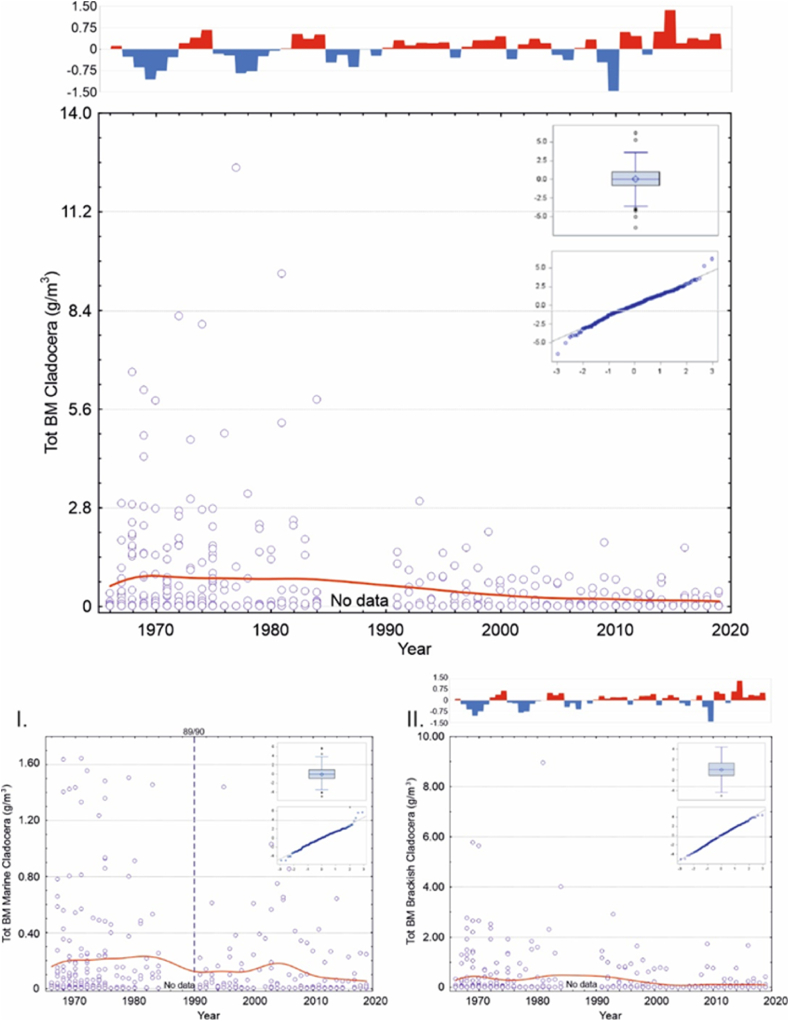
Top panel: Scatterplot for total Cladocera biomass (g m^−3^_wwt_) change at the sampling station in 1966–2019 (smooth red line is drawn with distance-weighted least squares method). Small panels within the figure express the Box plot and Quantile residuals distribution achieved in the GLIMMIX model (for a more detailed explanation see text and Table 6). Panels below indicate the same for the main functional groups of I. Marine Cladocera and II. Brackish Cladocera and, otherwise, explanations as in Fig. 2. In the top and in Panel II, the progress of the general winter NAO index is indicated with vertical bars (red = positive index and blue = negative index) according to Hurrell [22]. (For interpretation of the references to colour in this figure legend, the reader is referred to the Web version of this article.)

**Fig. 5:**
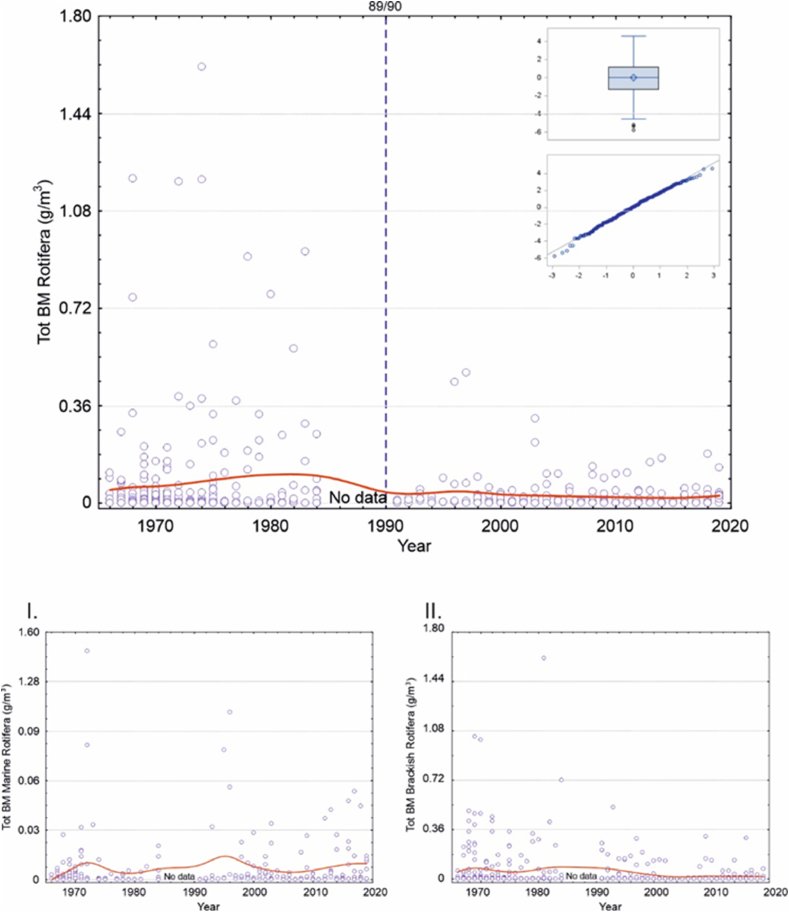
Top panel: Scatterplot for total Rotifera biomass (g m^−3^_wwt_) change at the sampling station from 1966 to 2019 (smooth red line is drawn with distance-weighted least squares method). Small panels within the figure express Box plot and Quantile residuals distribution of the achieved GLIMMIX model (for more detailed explanation see text and Table 7). Panels below indicate the same for the main functional groups of: I. Marine Rotifera and II. Brackish Rotifera and, otherwise, explanations as in Fig. 2. (For interpretation of the references to colour in this figure legend, the reader is referred to the Web version of this article.)

Our analyses revealed that for the groups: “total,” “marine,” and “brackish mesozooplankton”; “total,” “marine,” and “brackish copepods; ” and “marine cladocerans” and “total rotifers; ” the best model was obtained by using the RS as the only explanatory variable. Distinct regimes were classified as system changes in 1975/76 and 1989/90 (Fig. 2, Fig. 3, Fig. 4, Fig. 5, Table 3, Table 4, Table 6, Table 7). Moreover, the influence of RS on mesozooplankton biomasses was more obvious in marine mesozooplankton and total and marine copepods than the other functional groups, as with the former, both confirmed RS had consistently significant estimate values in the analyses. With regards to marine mesozooplankton and total and marine copepods models, the years after the 1975/76 RS retained significantly higher estimate values indicating that the biomasses of these groups were at their highest after the Major Baltic Inflow (MBI) in 1975/1976 and continued until the 1989/90 RS (Table 3, Table 4). For brackish copepods as well as marine cladocerans and total rotifers, the regulative effect on the species’ biomass decrease was evident only with the 1989/90 RS (Fig. 3, Fig. 4, Fig. 5, Table 4, Table 6, Table 7). Additionally, statistically, a significant intercept showed a trend in each of the groups.

**Table 3:** GLIMMIX model fixed effect results and estimates for different regimes (0 < 1976, 1 = 1976–1989, 2 = 1990–2019) for Total, Marine, and Brackish mesozooplankton taxa. Month and regime shifts (RS) were used as explanatory variables in the models. The regular Chi-Square/DF was 0.97 for the Total, 1.72 for the Marine, and 0.98 for the Brackish mesozooplankton models.

Total mesozooplankton μg m^−3^						Marine mesozooplankton μg m^−3^					Brackish mesozooplankton μg m^−3^				
*n* = 651						*n* = 574					*n* = 644				
Effect	Est.	SE	DF	*t*	*p*	Est.	SE	DF	*t*	*p*	Est.	SE	DF	*t*	*p*
**Intercept**	12.1	0.1	292.7	85.3	<0.0005	8.9	0.2	1.0	47.4	0.0134	12.4	0.3	1.0	46.2	0.0138
**Month**	.	.	4.1	.	<0.0001	.	.	2.5	.	0.0071	.	.	7.3	.	<0.0001
***Jan***	−3.2	0.4	2.2	−9.0	0.0091	−3.4	0.3	2.8	−12.4	0.0015	−3.0	0.6	1.5	−5.5	0.0576
***Feb***	−4.4	0.4	1.9	−10.4	0.0106	−4.2	0.3	1.5	−13.4	0.0140	−4.2	0.5	1.5	−9.3	0.0259
***Mar***	−4.9	0.4	2.4	−13.8	0.0022	−5.2	0.4	1.5	−14.5	0.0140	−4.8	0.3	3.0	−15.2	0.0006
***Apr***	−3.9	0.6	2.1	−6.5	0.0194	−5.1	0.6	2.4	−8.8	0.0067	−3.7	0.5	2.8	−7.5	0.0058
***May***	−2.3	0.4	2.4	−5.2	0.0235	−3.1	0.9	2.2	−3.4	0.0661	−2.2	0.3	4.0	−6.7	0.0026
***Jun***	0.1	0.4	2.4	0.2	0.8952	1.3	0.9	2.2	1.4	0.2894	−0.4	0.3	2.6	−1.3	0.2914
***Jul***	1.4	0.2	1.7	6.2	0.0352	2.1	0.5	2.4	4.6	0.0309	0.7	0.3	1.2	2.5	0.2165
***Aug***	0.9	0.2	1.4	3.8	<0.1066	0.5	0.3	2.3	1.4	0.2899	1.1	0.4	1.1	2.6	0.2222
***Sep***	0.0	.	.	.	.	0.0	.	.	.	.	0.0	.	.	.	.
***Oct***	−0.8	0.2	618.6	−4.5	<0.0001	−0.9	0.2	1.5	−4.1	0.0916	−0.7	0.4	1.0	−5.5	0.0576
***Nov***	−2.1	0.3	2.5	−7.8	0.0086	−2.7	0.4	1.7	−7.1	0.0275	−2.0	0.5	1.4	−4.0	0.1039
***Dec***	−2.5	0.3	2.7	−9.7	0.0034	−2.9	0.2	1.7	−12.3	0.0120	−2.4	0.5	1.4	−4.4	0.0913
**RS**	.	.	35.8	.	<0.0001	.	.	13.3	.	<0.0001	.	.	3.9	.	0.0138
***0 vs***. ***1***	0.2	0.2	40.7	1.3	0.2093	0.5	0.2	25.8	2.3	0.0317	0.0	0.2	7.0	0.3	0.8112
***0 vs***. ***2***	1.6	0.1	30.2	11.4	<0.0001	2.9	0.2	5.9	17.2	<0.0001	0.8	0.1	2.5	5.1	0.0219
***1 vs***. ***2***	1.4	0.2	39.4	7.8	<0.0001	2.4	0.2	25.1	11.4	<0.0001	0.7	0.2	5.2	4.1	0.0089

**Table 4:** GLIMMIX model fixed effect results and estimates for different regimes (0 < 1976, 1 = 1976–1989, 2 = 1990–2019) for Total, Marine, and Brackish Copepoda. Month and regime shifts (RS) were used as explanatory variables in the models. The regular Chi-Square/DF was 1.00 for the Total, 1.48 for the Marine, and 0.98 for the Brackish Copepoda models.

Total Copepoda μg m^−3^						Marine Copepoda μg m^−3^					Brackish Copepoda μg m^−3^				
*n* = 650						*n* = 510					*n* = 642				
Effect	Est.	SE	DF	*t*	*p*	Est.	SE	DF	*t*	*p*	Est.	SE	DF	*t*	*p*
**Intercept**	12.0	0.1	72.1	85.1	<0.0001	7.5	0.5	1.8	16.0	0.0054	11.8	0.1	222.5	83.3	<0.0001
**Month**	.	.	2.4	.	0.0057	.	.	4.4	.	0.0043	.	.	5.1	.	0.0003
***Jan***	−2.8	0.5	1.0	−5.3	0.1186	−2.0	0.5	2.4	−4.0	0.0410	−2.8	0.4	2.4	−7.4	0.0111
***Feb***	−4.1	0.2	628.6	−18.9	0.0001	−2.8	0.5	2.7	−5.3	0.0170	−4.1	0.4	2.0	−9.8	0.0098
***Mar***	−4.6	0.2	1.0	−20.3	0.0313	−3.8	0.7	3.1	−5.7	0.0101	−4.5	0.3	2.4	−13.4	0.0028
***Apr***	−3.4	0.7	1.0	−4.7	0.1328	−4.0	0.7	4.0	−6.0	0.0041	−3.6	0.6	2.1	−5.6	0.0277
***May***	−2.3	0.2	1.0	−13.1	0.0487	−2.8	0.7	4.0	−3.9	0.0177	−2.3	0.3	2.6	−8.1	0.0065
***Jun***	−1.0	0.5	1.0	−1.9	0.3075	−1.3	0.7	3.8	−1.9	0.1417	−1.3	0.5	2.1	−2.4	0.1337
***Jul***	0.3	0.2	628.1	1.7	0.0866	−0.1	0.5	2.2	−0.2	0.8881	0.3	0.2	3.1	1.6	0.2035
***Aug***	0.1	0.3	1.0	0.2	0.8702	0.1	0.6	2.4	0.3	0.8136	0.1	0.2	1.1	0.6	0.6412
***Sep***	0.0	.	.	.	.	0.0	.	.	.	.	0.0	.	.	.	.
***Oct***	−0.6	0.3	1.0	−2.3	0.2641	−0.1	0.6	2.8	−0.2	0.8513	−0.6	0.2	610.5	−3.6	0.0004
***Nov***	−1.7	0.4	1.0	−4.4	0.1410	−1.4	0.6	3.2	−2.4	0.0932	−1.8	0.3	2.6	−6.2	0.0127
***Dec***	−2.3	0.5	1.0	−4.8	0.1322	−1.6	0.5	2.2	−3.2	0.0763	−2.2	0.3	2.3	−7.3	0.0119
**RS**	.	.	7.0	.	<0.0001	.	.	60.4	.	<0.0001	.	.	26.7	.	<0.0001
***0 vs***. ***1***	0.3	0.1	623.0	2.9	0.0035	0.4	0.2	62.9	2.0	0.0454	0.2	0.2	39.3	1.2	0.2548
***0 vs***. ***2***	1.3	0.1	4.6	10.9	0.0002	2.9	0.2	62.4	15.6	<0.0001	1.4	0.1	16.5	10.8	<0.0001
***1 vs***. ***2***	1.0	0.1	8.8	6.8	<0.0001	2.4	0.2	55.9	10.9	<0.0001	1.2	0.2	38.4	7.4	<0.0001

Similarly, when monthly estimates were compared against the September control value, although not always significant, the higher values were almost invariably found during the growing season (i.e., June to September) indicating normal yearly life cycles of these groups. The negative value of a month indicated a smaller biomass compared to September (Table 3, Table 4, Table 5, Table 6, Table 7).

For limnic copepods, the model showed that the most important variable explaining the biomass change was the number of ice days indicating the influence of a shortened ice cover period on the annual seawater temperatures and further on the cool-water affiliated species since the 1960s (Table 5). The results are the same for limnic mesozooplankton, as this functional group solely consists of the copepod *L*. *macrurus*. The same was revealed by another classifying variable, “10-year intervals,” of which, only the first ten years (1966*–*1975) indicated higher biomasses when compared to the other decades (Table 5). Here, as well, the intercept revealed a trend in series, but between the months, the life-cycle seasonality was not apparent.

**Table 5:** GLIMMIX model fixed effect results for Limnic Copepoda. Month, 10-year intervals (Intervals), and number of ice days were used as explanatory variables in the model. The regular Chi-Square/DF for the model was 1.53.

Limnic Copepoda μg m^−3^					
*n* = 202					
Effect	Est.	SE	DF	*t*	*p*
**Intercept**	5.2	0.6	36.3	8.3	<0.0001
**Month**	.	.	12.5	.	0.0106
***Jan***	−0.4	0.6	23.3	−0.8	0.4518
***Feb***	−2.4	0.6	23.1	−4.1	<0.0004
***Mar***	−2.0	0.6	20.2	−3.5	0.0021
***Apr***	−1.0	0.5	16.2	−1.8	0.0841
***May***	−0.8	0.6	25.6	−1.4	0.1610
***Jun***	−1.2	0.7	17.3	−1.6	0.1328
***Jul***	0.5	1.1	9.5	0.5	0.6433
***Aug***	−0.0	0.8	20.3	−0.0	0.9684
***Sep***	0.0	.	.	.	.
***Oct***	−0.4	0.7	17.0	−0.6	0.5434
***Nov***	−0.7	0.6	26.3	−1.2	0.2595
**Intervals**	.	.	35.2	.	0.0033
***66****–****75***	1.5	0.5	35.4	3.3	<0.0023
***76****–****85***	0.1	0.8	18.4	0.1	0.9306
***86****–****95***	0.8	0.7	65.2	1.1	0.2857
***96****–****05***	0.3	0.5	69.9	0.7	0.4978
***06****–****15***	0.4	0.4	69.8	0.8	0.4239
***16****–****19***	0.0	.	.	.	.
**Ice days**	0.0	0.0	64.9	2.9	0.0045

For cladocerans, as well, most of the total biomass was composed of brackish water species, and the marine taxa represented only a minority. Similarly, there seemed to be a biomass decrease between 1984 and 1991 (Fig. 4, Table 6).

**Table 6:** GLIMMIX model fixed effect results for the Total, Marine, and Brackish Cladocera models. Month and Winter NAO produced the most parsimonious model for Total Cladocera, while month and regime shifts (RS; 0 < 1976, 1 = 1976*–*1989, 2 = 1990–2019) were used as explanatory variables for Marine Cladocera. Month, 10-year intervals (Intervals), and Winter NAO best explained the changes in Brackish Cladocera. The regular Chi-Square/DF was 1.78 for the Total, 1.76 for the Marine, and 1.89 for the Brackish Cladocera models.

Total Cladocera μg m^−3^						Marine Cladocera μg m^−3^					Brackish Cladocera μg m^−3^				
*n* = 420						*n* = 344					*n* = 391				
Effect	Est.	SE	DF	*t*	*p*	Est.	SE	DF	*t*	*p*	Est.	SE	DF	*t*	*p*
**Intercept**	10.4	0.4	42.3	30.0	<0.0001	9.3	0.2	52.9	37.2	<0.0001	9.6	0.5	125.2	19.4	<0.0001
**Month**	.	.	46.5	.	<0.0001	.	.	1.0	.	0.0975	.	.	12.9	.	<0.0001
***Jan***	−3.6	0.5	7.1	−6.6	0.0003	−4.8	1.7	330.0	−2.9	0.0044	−3.7	0.5	140.1	−7.0	<0.0001
***Feb***	−4.3	0.6	161.8	−7.6	<0.0001	−5.9	1.1	1.2	−5.5	0.0879	−4.5	0.5	142.5	−8.4	<0.0001
***Mar***	−3.8	0.6	3.1	−6.3	0.0075	−4.7	1.6	1.1	−3.0	0.1788	−4.4	0.6	23.4	−7.7	<0.0001
***Apr***	−3.8	0.5	25.0	−7.2	<0.0001	−5.2	0.4	30.6	−12.9	<0.0001	−4.7	0.5	114.4	−9.8	<0.0001
***May***	−2.1	0.4	70.6	−5.0	<0.0001	−2.2	0.4	42.3	−5.0	<0.0001	−2.4	0.4	64.4	−5.7	<0.0001
***Jun***	1.8	0.4	67.5	4.4	<0.0001	1.9	0.3	76.7	6.0	<0.0001	−1.2	0.4	73.3	−2.7	<0.0090
***Jul***	3.2	0.4	66.2	7.9	<0.0001	2.2	0.3	65.8	7.4	<0.0001	1.8	0.5	76.9	3.9	<0.0002
***Aug***	2.5	0.4	72.7	5.8	<0.0001	0.4	0.3	58.9	1.2	0.2504	2.8	0.4	60.5	6.6	<0.0001
***Sep***	0.0	.	.	.	.	0.0	.	.	.	.	0.0	.	.	.	.
***Oct***	−1.5	0.4	64.9	−3.4	<0.0012	−1.5	0.3	40.5	−4.9	<0.0001	−1.6	0.5	50.9	−3.6	0.0007
***Nov***	−3.5	0.5	30.1	−7.6	<0.0001	−3.8	0.4	11.7	−9.3	<0.0001	−3.7	0.5	10.0	−8.0	<0.0001
***Dec***	−4.1	0.8	6.2	−5.3	0.0016	−4.7	1.0	146.6	−4.9	<0.0001	−3.8	0.7	5.4	−5.3	0.0024
**Intervals**											.	.	211.7	.	<0.0001
***66****–****75***											0.4	0.4	209.8	0.9	0.3522
***76****–****85***											0.7	0.4	283.3	1.7	0.0963
***86****–****95***											1.5	0.5	246.7	2.9	0.0045
***96****–****05***											−0.5	0.4	272.6	−1.1	0.2766
***06****–****15***											−0.5	0.4	276.8	−1.3	0.1991
***16****–****19***											0.0	.	.	.	.
**Winter NAO**	−0.46	0.17	193.3	−2.7	0.0068						−0.5	0.2	169.2	−2.5	0.0137
**RS**						.	.	186.3	.	<0.0001					
***0 vs***. ***1***						−0.3	0.3	187.4	−1.0	0.3224					
***0 vs***. ***2***						1.5	0.2	156.7	7.6	<0.0001					
***1 vs***. ***2***						1.7	0.3	258.7	6.6	<0.0001					

The best models obtained for total and brackish cladocerans biomass changes were those in which the explanatory variable was the winter NAO indicating that, during the first ten-year period of 1966–1975, biomasses were higher than when compared with the rest of the decades (Table 6). A statistically significant winter NAO proved that the areal temperature rise and the increased precipitation into the Baltic catchment area or the interaction of them both had a negative influence on cladocerans in general. Again, for marine cladocerans, the most important explanatory variable was RS, but here, only the latter shift of 1989/90 showed a significant effect. Here, as well, the intercept revealed a trend in series. Similarly, when compared with marine copepods, when monthly estimates were compared against the September control value, there was a clear indication that higher values were invariably found during the growing season being June to September indicating normal yearly life cycles of this species group (Table 6).

Again, with rotifers, most of the total biomass was composed of brackish water species. Similarly, there seemed to be a biomass decrease between 1984 and 1991, but, here, it was not so obvious depending on the functional trait (Fig. 5).

With rotifers, the results were similar to those of cladocerans, however, we considered that the models were not very plausible. We found a moderate model only for the total rotifer's biomass changes, which included a significant effect in the 1989/90 RS (Table 7). Between the months, the life-cycle seasonality was not apparent for these groups (Table 7).

**Table 7:** GLIMMIX model fixed effect results for Total Rotifera. Month and regime shifts (RS; 0 < 1976, 1 = 1976–1989, 2 = 1990–2019) were used as explanatory variables for the model. The regular Chi-Square/DF for the model was 2.07.

Total Rotifera μg m^−3^					
*n* = 374					
Effect	Est.	SE	DF	*t*	*p*
**Intercept**	8.4	0.3	50.0	31.6	<0.0001
**Month**	.	.	5.6	.	<0.0001
***Jan***	−4.5	0.8	6.1	−5.5	0.0014
***Feb***	−7.0	0.6	3.3	−10.9	<0.0011
***Mar***	−6.7	0.7	1.7	−9.6	0.0172
***Apr***	−3.9	0.6	19.8	−6.9	<0.0001
***May***	−1.2	0.5	50.7	−2.6	0.0135
***Jun***	2.3	0.4	75.8	5.9	<0.0001
***Jul***	1.4	0.3	71.0	4.0	0.0002
***Aug***	0.4	0.4	69.5	1.2	0.2517
***Sep***	0.0	.	.	.	.
***Oct***	−0.0	0.4	70.3	−0.0	0.9729
***Nov***	−2.8	0.4	69.6	−7.4	0.0001
***Dec***	−5.3	0.6	23.7	−9.0	<0.0001
**RS**	.	.	193.4	.	0.0029
***0 vs***. ***1***	−0.3	0.3	181.8	−1.1	0.2899
***0 vs***. ***2***	0.5	0.2	137.6	2.1	0.0393
***1 vs***. ***2***	0.8	0.2	240.2	3.2	0.0014

## 4 Discussion

This study brings forth evidence of a biomass and functional biodiversity loss amongst mesozooplankton of the northern Baltic Sea. Our results show, for the first time, the impact of two climate-driven RS for the Archipelago Sea mesozooplankton community during the time frame from 1966 to 2019. We found that the confirmed RS in 1975 and 1976 and in 1989 and 1990 explained the best the observed variation of trends in the local mesozooplankton communities. This is a good example of the regulative effects of large-scale climatic anomalies on mesozooplankton biomass changes in the Northern Baltic Sea. Although the effect was obvious in most of the functional groups, it was more predominant with marine mesozooplankton showing a two-way regulative effect during different eras. Between 1976 and 1990, the RS favored marine mesozooplankton when their biomasses were at their highest. The reason for this is understandable, as the 75/76 MBI from the North Sea through the Danish Straits into the Baltic Sea was the third largest barotropic inflow in the known MBI records [46]. The salinity change is visible with a small lag. However, the salinity started to decrease already from the late 1970s because of a lack of new inflow events [47] and increasing freshwater runoff [6,46]. This was reflected in the marked decrease of marine mesozooplankton, copepods, and marine copepods such as *T*. *longicornis* [13,48,49]. Our current study forms a continuum with the study by Hänninen et al. [15]. We speculate that they were unveiling the same functional biodiversity loss caused by changes in the Atlantic climatic factors.

The latter RS was caused by a change in the winter NAO into a predominantly positive mode from the end of the 1980s [23]. There is evidence of a link between climate change and the shift in the winter NAO, as most of the climate models indicate a connection between increased amounts of tropospheric greenhouse gases and positive winter NAO values [50].

We found that the biomasses of the studied mesozooplankton groups correlated negatively with the winter NAO, which is opposite to the findings of Hansson et al. [51]. The results of the forementioned study can be explained by the relatively short time frame of 13 years compared to a time frame of over 50 years in this study. Furthermore, the impact of seasonal mesozooplankton phenology was not included in the results of Hansson et al. [51], whereas the impact of a seasonal autocorrelation into biomasses was included in our results.

The decrease of marine and brackish copepods can be seen as a parallel biomass decrease of both groups, though marine mesozooplankton have suffered the most. The biomass of the limnic copepod *L*. *macrurus*, being the sole representative of freshwater mesozooplankton in this study, has also decreased from the 1966 levels in the uppermost 25 m. However, as indicated by a study of Rajasilta et al. [52], *L*. *macrurus* is currently an abundant species in the Bothnian Sea. The species has most likely migrated into deeper water layers because of rising temperatures, as it is a cold-water stenotherm [40,52]. The length of the ice season has, in general, decreased in the northern Archipelago Sea affecting the optimum spatial position for the species in the water column.

According to our results, the biomasses of total and brackish cladocerans have decreased between 1984 and 1991 responding to the predominantly positive shift in the winter NAO. The biomass of marine cladocerans best responded to the latter RS event and started decreasing since. Using the SEILI mesozooplankton abundance series between the time frame of 1967–2013, Mäkinen et al. [37] found that the abundances of marine cladocerans: *E*. *nordmanni*, *P*. *polyphemoides*, and *Podon* spp. decreased significantly. According to our results, the biomass trends for the marine cladocerans: *E*. *nordmanni*, *P*. *polyphemoides*, and *Podon* spp. matched the abundance trends found in Mäkinen et al. [37]. Further, according to Mäkinen et al. [37], the biomass anomaly of the brackish species *B*. *longispina maritima* was negative during 2000–2013, but the negative trend was not significant, as the anomaly had been positive during 1991–2000. We found that the biomass decrease for the brackish cladoceran *B*. *longispina maritima* was significant during the time frame from 1966 to 2019. Furthermore, we proved that the shift in the winter NAO starting during the end of the 1980s, which induced a RS, is behind the observed biomass decreases for brackish cladocerans. It is also possible that *C*. *pengoi* had a minor influence on the zooplankton community, as *B*. *longispina maritima* abundances decrease simultaneously with the sharp *C*. *pengoi* abundance increase in the Gulf of Riga [53]. However, the biomass of *C*. *pengoi* in the samples was small since its arrival in 1998. The species appeared in them mainly during summer and autumn. During most years, its biomasses were between 0 and 50 mg m^−3^, although in 2014 and 2017, it reached biomasses of 200*–*250 mg m^−3^. Hence, it is unlikely that *C*. *pengoi* has had a significant effect on the zooplankton community of the Archipelago Sea.

Similarly with other mesozooplankton groups, Hansson et al. [51] found that also the abundance of cladocerans correlated positively with the winter NAO. Above, we have explained the reasons for the differing results with Hansson et al. [51]. Furthermore, Möllmann et al. [14] found that cladocerans correlated positively with temperature. The former studies were between 1959 and 1997 [14] and between 1976 and 1998 [51] thus lacking the nearly two decades from the beginning of the 21st century, which are included in our study. In light of several studies, it becomes evident that significant developments have occurred in the marine ecosystems since the turn of the millennium as a response to environmental forcing [e.g. Refs. [3,18,54]].

The biomasses of total rotifers, which have slightly decreased since the beginning of the study period, responded best to the latter RS. However, when observing the biomasses of marine and brackish rotifers separately, there appeared to be no major population changes. Earlier, it has been shown that climate warming favors rotifers, in general. In separate studies, higher abundances of *Synchaeta* spp. have been observed after milder winters [37,55,56]. However, a salinity decrease related to the second RS has had a negative effect on rotifers, as especially *S*. *monopus* is a marine species with an affinity to higher salinities [57]. According to our results, the recent changes in large-scale climatic factors have been detrimental to rotifers as well. However, we were not able to detect significant changes in the biomass of *S*. *monopus* probably due to the small number of specimens in the samples (model n = 169). Instead, for rotifers, the biomass decline could only be assessed for the *Synchaeta* spp. level (model n = 374), and it was not as clear as for cladocerans and copepods.

We found that the biomasses of marine copepods decreased the most, but also brackish copepods reduced significantly. Mäkinen et al. [37] detected decreased abundances for marine and large-bodied mesozooplankton species in the Archipelago Sea, while in their study, they did not find a clear abundance trend for brackish water species. Further, their study indicated increases in the abundance of small-sized mesozooplankton species, but at a rather robust level, as they combined several functional taxa including small copepods, cladocerans, and rotifers. Our study found decreasing biomass trends for both the marine and brackish mesozooplankton starting already from the end of the 1970s. The change in mesozooplankton biomasses occurred synchronously with a surface water salinity decrease and a temperature increase [e.g., 37], which are ultimately regulated by large-scale climatic factors [15]. Our study did not confirm the results that a warmer and less saline environment would favor smaller mesozooplankton. Although this might be affected by the fact that *Keratella* spp. rotifers were not included because they were smaller than the used plankton net (APPENDIX). Viitasalo and Bonsdorff [57] pointed out that especially some cladoceran and rotifer species expressed better adaptability to warmer temperatures. Due to the changed salinity conditions in the Northern Baltic Sea, the winners of the mesozooplankton community most probably include freshwater-originated cladoceran and rotifer species with affinities to warmer water temperatures [13,58]. Indeed, using the Seili mesozooplankton series, we can confirm that the freshwater-originated cladoceran species, *D*. *cucullata* (Sars), has increased in abundances in the Archipelago Sea since the end of the 1990's, and during some years, has reached peak abundances of up to 350 individuals m^−3^. However, the energetic value of cladocerans, as feed for Baltic herring (*Clupea harengus membras* Linnaeus, 1760; from here on referred to as herring), is poorer than that of copepods [59].

Although the study only includes 12 of the 65 observed taxa in the samples during the study period, these included taxa represent the majority of the total mesozooplankton biomass. The excluded taxa are mostly relatively insignificant regarding the total mesozooplankton biomass, or they could not be quantitatively sampled (APPENDIX; see full explanation in the Material and Methods section).

Possible changes in sampling efficiency due to the amendment in the mouth diameter are previously discussed by Vuorinen et al. [13]. Due to the geometry and length-width ratios of the nets used, the sampling efficiency should have increased after the change of nets cf.[60].

There is a gap in the data series during 1985*–*1990, because of temporary closing of the plankton monitoring program. While we acknowledge that we are unaware of the mesozooplankton biomasses from that 5-year period, it is unlikely that this has an impact on the results and conclusions drawn. This is because the biomasses were already decreasing from the late 1970's to 1984, and in 1991, when the sampling program continued, the biomasses were significantly lower than in 1984.

Previously, several studies have detected abrupt changes in the Baltic Sea ecosystem [e.g. Refs. [7,18,26]]. However, this is the first study that validates the 1975/76 RS in the Baltic Sea with mesozooplankton, as major modifications in zooplankton linked to the RS in question have previously been confirmed in the Pacific Ocean [2 and references therein]. The late 1970s RS followed an abrupt change in the large-scale boreal winter circulation pattern over the North Pacific in the mid-1970s affecting the thermal regime of the oceanic regions [2]. The RS for 1976 and 1990 have been detected quasi-synchronously globally in marine ecosystems [e.g. Refs. [2,7]]. Teleconnections by Arctic Oscillation (AO), Atlantic multidecadal oscillation (AMO), North Atlantic Oscillation (NAO), and Pacific decadal oscillation (PDO) among world oceanic systems mediate recorded stepwise temperature and salinity changes related to the RS [2,61]. The effect of a temperature increase on zooplankton is further magnified through the impact of hydrographic changes on phytoplankton populations and their phenology [e.g. Refs. [2,23,26]].

An alternative hypothesis states that changes in the food web of the Baltic Sea were behind the drastic declines of important prey species for herring, *P*. *acuspes* and *T*. *longicornis* [e.g. Refs. [16,62]]. Important zooplanktivores in the Baltic Sea ecosystem include herring, sprat (*Sprattus sprattus* Linnaeus, 1758), and mysids, such as *Mysis mixta* Liljeborg, 1853 and *Neomysis integer* Leach, 1814 [e.g. Refs. [16,62]]. This theory explains the changes in zooplankton community structure by a top-down control of clupeids and mysids [16]. Sprat has greatly increased in abundance since the late 1980s coinciding with changes in the abiotic environment and the decline of cod [16,26,61].

On the contrary, Möllmann and Köster [63] did not find evidence of a strong top-down control by clupeids on mesozooplankton, but instead concluded that bottom-up forces, such as hydrographic changes and changes in food resources, were behind the observed changes in mesozooplankton communities. In line with the forementioned study, our findings support the earlier results stating that, ultimately, the observed changes in zooplankton species biomasses are caused by climatic forcing. Thus, we confirm the results by others, [e.g. Refs. [15,29,64]], that climate variability-induced abiotic changes in the marine environment strongly affect the mesozooplankton species. Bradley and Ormerod [65] detected instability in stream invertebrate communities in Wales during the positive phases of the winter NAO. Similarly, we found that the positive phases of the winter NAO negatively affect mesozooplankton communities in the Baltic Sea.

Here, we demonstrate a considerable biomass and functional biodiversity loss of the common mesozooplankton, the primary consumers of the Archipelago Sea from the 1966 until 2019, in relation to climatic forcing. The obtained results are important, as we show the impact of large-scale climatic anomalies on mesozooplankton. Further, we add to the existing knowledge by showing that, besides the RS in 1990, also the 1976 RS was significant for the mesozooplankton of the northern Baltic Sea. Furthermore, we brought forth evidence that also the common cladocerans have suffered due to a NAO-mediated temperature increase and a salinity decrease. The findings of this study have important implications for planktivorous fish, such as the herring [52]. During previous decades, herring mostly fed on large marine mesozooplankton species, such as *P*. *acuspes* and *T*. *longicornis* [1]. Recently, the fish have partly replaced the large marine species in its diet with *L*. *macrurus* in the Bothnian Sea, where this limnic copepod is abundant below 25 m [52]. However, herring biomass and lipid reserves have decreased significantly from the 1980s [52,66]. This has already led to a management-level debate about the sustainability of herring fishery and limitations, which even led to a proposed ban on fishing (Commission proposal fishing opportunities Baltic Sea 2024). As the effects of climate change on marine ecosystem increase, it is likely that species adapted to live in high salinity or cold temperature environments are largely replaced by freshwater species with affinities to warmer water, such as the cladocerans *D*. *cucullata* and *C*. *pengoi* [1,53]. In this respect, the Baltic Sea may even function as a “time machine,” as proposed by Reusch et al. [3], to predict the impact of future climate change on marine ecosystems in comparable sea areas. Importantly, Baltic Sea temperatures are predicted to increase by 2*–*4°C by the end of the century [67].

## Data availability

Data is included in the article/supporting material/referenced in article.

## CRediT authorship contribution statement

**Heta Rousi:** Writing – review & editing, Writing – original draft, Visualization, Validation, Methodology, Investigation, Funding acquisition, Formal analysis, Data curation, Conceptualization. **Julia Fält-Nardmann:** Writing – review & editing, Writing – original draft, Visualization, Investigation, Funding acquisition, Conceptualization. **Pekka Niemelä:** Writing – review & editing, Writing – original draft, Supervision, Investigation, Funding acquisition, Conceptualization. **Jari Hänninen:** Writing – review & editing, Writing – original draft, Visualization, Validation, Supervision, Project administration, Methodology, Investigation, Funding acquisition, Formal analysis, Data curation, Conceptualization.

## Declaration of competing interest

The authors declare that they have no known competing financial interests or personal relationships that could have appeared to influence the work reported in this paper.

## Figures and Tables

**Table:** 

Taxon	Ecological group	Present in % samples	% of total biomass	length (mm)	Year of occurrence
***Acartia* sp**. ***adult***	**Copepod (holoplankton)**	**95.00**	**9.00**	**0.90**–**1.50**	**1966**–**2019**
***Acartia* sp**. **CI-CV**	**Copepod (holoplankton)**	**98.90**	**12.55**	**0.50**–**0.90**	**1966**–**2019**
*Acartia* sp. *nauplii*	Copepod (holoplankton)	97.40	0.11	0.07–0.10	1966–2019
*Amphibalanus improvisus* juv.	Barnacle (meroplankton)	1.10		0.50	1991, 1994, 1996, 2005, 2010
*Amphibalanus improvisus* nauplii	Barnacle (meroplankton)	28.80		0.43	1976–1984, 1991–2019
Bivalvia larva	Bivalve (meroplankton)	28.30		0.09–0.40	1976–1984, 1991–2019
***Bosmina longispina maritima***	**Cladoceran (holoplankton)**	**61.00**	**25.11**	**0.40**–**1.20**	**1966**–**2019**
Bryozoa	Bryozoan (sedentary, colonial)	0.10		0.50	2013
Calanoida adult	Copepod (holoplankton)	0.30		0.50–2.90	2007, 2012
Calanoida CI-CV	Copepod (holoplankton)	18.60		0.20–1.90	1991–2014, 2016–2017, 2019
Calanoida nauplii	Copepod (holoplankton)	8.90		0.06–0.60	1991–1993, 1995, 1997, 1999–2000, 2005–2006, 2008–2014, 2016–2019
***Centropages hamatus* adult**	**Copepod (holoplankton)**	**39.85**	**0.87**	**1.20**–**1.60**	**1966**–**1984, 1992**–**1993, 1995**–**2005, 2007**–**2019**
***Centropages hamatus* CI-CV**	**Copepod (holoplankton)**	**48.20**	**0.85**	**0.05**–**1.10**	**1966**–**1994, 1995**–**2019**
Centropages hamatus nauplii	Copepod (holoplankton)	12.90	0.01	0.03–0.05	1966–1994, 1995–1997, 2003–2005, 2007–2019
*Cercopagis pengoi*	Cladoceran (holoplankton)	4.70	0.15	6.00–13.0_1_	1998–2002, 2008–2012, 2014–2019
*Chydorus* sp.	Cladoceran (holoplankton)	0.80		0.20–0.50	1999, 2009, 2012, 2018–2019
Copepoda adult	Copepod (holoplankton)	10.20		1.00–2.00	1996–2008, 2017
Copepoda CI-CV	Copepod (holoplankton)	1.40		0.40–1.90	2005, 2007–2008, 2012
Copepoda nauplii	Copepod (holoplankton)	11.50		0.05–0.50	1996–2008, 2012, 2014,
Copepoda ova	Copepod (holoplankton)	0.30		0.05–0.09	2007
*Cothurnia* sp.	Ciliophore (mostly sessile)	0.90		0.05	2017
Cyclopidae adult	Copepod (holoplankton)	5.30		0.50–2.00	1991–1992, 1993, 1995–1996, 1999, 2001–2004, 2008, 2011–2015, 2017
Cyclopidae CI-CV	Copepod (holoplankton)	23.80		0.40–1.90	1991–2017
Cyclopidae nauplii	Copepod (holoplankton)	7.00		0.05–0.50	1991–1996, 2003–2007, 2013–2019
Cyclopinae CI-CV	Copepod (holoplankton)	2.60		0.40–1.90	2015, 2018–2019
*Cyclops* sp. adult	Copepod (holoplankton)	0.90		0.50–2.00	2007, 2010, 2012, 2014
*Cyclops* sp. CI-CV	Copepod (holoplankton)	1.10		0.40–1.90	1996, 2000, 2003, 2007, 2014
*Cyclops* sp. nauplii	Copepod (holoplankton)	1.70		0.05–0.50	1999–2000, 2002, 2005, 2009–2010, 2012
*Daphnia* spp. adult	Cladoceran (holoplankton)	4.40		1.00–5.00	1966–1975, 2012, 2014–2015, 2017–2018
*Daphnia* spp. juv.	Cladoceran (holoplankton)	0.90		0.20	2012, 2014–2015, 2018
*Daphnia cristata* adult	Cladoceran (holoplankton)	0.30		0.60–1.80	2018
*Daphnia cristata* juv.	Cladoceran (holoplankton)	0.50		0.20	2017–2019
*Daphnia cucullata* adult	Cladoceran (holoplankton)	5.60		0.60–1.70	1992, 1994-1995-1999, 2001–2016
*Daphnia cucullata* juv.	Cladoceran (holoplankton)	2.70		0.20–0.40	1992, 1995, 1997–1998, 2000, 2003, 2009–2010, 2013–2016
Decapoda zoea larvae	Decapod (meroplankton)	0.60		0.50–20.0	2018–2019
*Diacyclops* sp. adult	Copepod (holoplankton)	3.00		0.90–1.60	1994–1997, 1999–2004, 2007–2008, 2011
*Diacyclops* sp. CI-CV	Copepod (holoplankton)	4.70		0.40–1.50	1994–2001, 2003–2007
*Didinium* sp.	Ciliate (planktonic)	0.20		0.05–0.15	2016
*Einhornia crustulenta*	Bryozoan (attached, colonial)	4.90		0.50_2_	2011–2019
***Eurytemora affinis adult***	**Copepod (holoplankton)**	**90.30**	**4.62**	**1.20**–**1.30**	**1966**–**2019**
***Eurytemora affinis CI-CV***	**Copepod (holoplankton)**	**95.45**	**8.73**	**0.50**–**1.10**	**1966**–**2019**
*Eurytemora affinis nauplii*	Copepod (holoplankton)	59.70	0.02	0.10–0.40	1996–1996, 1998–2002, 2004–2005, 2008–2019
*Evadne anonyx*	Cladoceran (holoplankton)	0.50		0.65–1.08	2015–2017
***Evadne nordmanni***	**Cladoceran (holoplankton)**	**38.50**	**2.97**	**0.50**–**0.90**	**1966**–**2019**
*Fritillaria borealis*	Tunicate (planktonic)	1.10		4.00	2001, 2008, 2011, 2014, 2016, 2018
Gastropoda juv.	Gastropod (meroplankton)	6.70		0.10–0.20	1991–1996, 2009–2017
Harpacticoida adult	Copepod (holoplankton)	18.90		0.20–2.50	1991, 1995, 1997–2019
Harpacticoida juv.	Copepod (holoplankton)	2.60		0.05–0.10	2011–2013, 2015–2018
*Helicostomella* sp.	Ciliate (planctonic)	0.60		0.20–0.50	2010, 2011, 2013
*Helicostomella subulata*	Ciliate (planctonic)	0.60		0.20–0.50	2014, 2017, 2019
*Keratella* spp.	Rotifer (holoplankton)	21.80		0.10–0.40	1966–1975, 1998–1999
*Keratella quadrata*	Rotifer (holoplankton)	71.80		0.27–0.33	1966–1985, 1991–2019
*Keratella quadrata platei*	Rotifer (holoplankton)	2.00		0.20–0.30	1999, 2009–2011, 2014–2015, 2017
*Keratella cochlearis*	Rotifer (holoplankton)	9.10		0.10–0.20	1991, 1996–1998, 2000–2006, 2008–2019
*Keratella cochlearis recurvispina*	Rotifer (holoplankton)	1.20		0.10–0.20	1997–1998, 2003, 2009, 2013
*Keratella cruciformis*	Rotifer (holoplankton)	2.90		0.10–0.20	1993, 2001–2002, 2004, 2011–2018
*Leprotintinnus* sp.	Ciliate (planktonic)	1.20		0.15–0.20	2012–2013, 2018–2019
*Leprotintinnus bottnicus*	Ciliate (planktonic)	0.90		0.15–0.20	2014–2017
*Leptodora kindtii*	Cladoceran (holoplankton)	2.10		6.00–12.0	1998–2000, 2003, 2005–2007, 2009, 2013–2014, 2016, 2018
***Limnocalanus macrurus* adult**	**Copepod (holoplankton)**	**9.20**	**0.09**	**2.00**–**3.00**	**1966**–**1972, 1974**–**1975, 1984, 1992, 1994, 1996**–**2003, 2005, 2007, 2010**–**2014, 2015**
***Limnocalanus macrurus* CI-CV**	**Copepod (holoplankton)**	**31.70**	**0.13**	**1.00**–**2.00**	**1966**–**1970, 1972**–**1975, 1982, 1991**–**2019**
*Limnocalanus macrurus* nauplii	Copepod (holoplankton)	38.30	0.01	0.20–0.90	1966–1975, 1982–2019
*Marenzelleria neglecta*	Polychaete (meroplankton)	14.50		1.00–115.0	1991–2014
*Marenzelleria* spp. larvae	Polychaete (meroplankton)	5.00		1.00	2015–2019
*Marenzelleria* trochophore	Polychaete (meroplanton)	0.90		0.30	2016–2019
*Mesocyclops leuckarti*	Copepod (holoplankton)	0.20		1.00–1.30	2018
Mysidae	Mysid (semibenthic)	12.70		2.00–30.00	1996–2009, 2013–2019
*Notholca* sp.	Rotifer (holoplankton)	0.50		0.10–0.40	2012, 2017, 2019
***Pleopsis polyphemoides***	**Cladoceran (holoplankton)**	**47.30**	**28.40**	**0.30**–**0.70**	**1966**–**2019**
***Podon* sp**.	**Cladoceran (holoplankton)**	**17.90**	**0.16**	**0.90**–**1.20**	**1966**–**1977, 1979**–**2004, 2009**–**2018**
Polychaeta	Polychaete (meroplankton)	1.40		1.00–115.0	2012–2013
Polychaeta larvae	Polychaete (meroplankton)	1.80		1.00	2000, 2003, 2011, 2013–2014
***Pseudocalanus acuspes* adult**	**Copepod (holoplankton)**	**3.60**	**0.02**	**0.85**–**2.15**	**1966, 1968**–**1969, 1970**–**1971, 1973, 1977**–**1984**
***Pseudocalanus acuspes* CI-CV**	**Copepod (holoplankton)**	**15.45**	**0.02**	**0.50**–**0.80**	**1966**–**1975, 1977**–**1984, 1997**–**1998, 2006, 2010**–**2011, 2013**–**2014**
*Pseudocalanus acuspes* nauplii	Copepod (holoplankton)	0.60	0.00	0.20–0.40	**1966, 1971, 1973**
*Radiosperma* spp.	Ciliate (planktonic)	2.70		0.10–0.15	1993, 1999–2000, 2003–2006, 2008, 2010–2014, 2016
*Radiosperma corbiferum*	Ciliate (planktonic)	0.90		0.10–0.15	2015, 2018–2019
Rotatoria	Rotifer (holoplankton)	9.50		0.00–0.45	1991, 1997–2008, 2011
*Synchaeta* spp.	Rotifer (holoplankton)	12.60		0.15–0.25	1993–2002, 2004–2006, 2008–2019
***Synchaeta baltica***	**Rotifer (holoplankton)**	**68.50**	**5.22**	**0.35**–**0.50**	**1966**–**2019**
*Synchaeta curvata*-*fennica*	Rotifer (holoplankton)	0.20		0.15–0.25	2016
***Synchaeta monopus***	**Rotifer (holoplankton)**	**33.20**	**0.38**	**0.20**–**0.22**	**1966**–**1982, 1984**–**2019**
***Temora longicornis* adult**	**Copepod (holoplankton)**	**28.20**	**0.16**	**0.90**–**1.20**	**1966**–**1975, 1977**–**2019**
***Temora longicornis* CI-CV**	**Copepod (holoplankton)**	**37.90**	**0.27**	**0.50**–**0.80**	**1966**–**2019**
*Temora longicornis nauplii*	Copepod (holoplankton)	27.00	0.00	0.20–0.40	**1966**–**1984, 1993, 1998**–**2000, 2009, 2012, 2017, 2019**
*Thermocyclops dybowskii* adult	Copepod (holoplankton)	1.10		0.60–1.00	2016–2019
*Thermocyclops dybowskii* CI-CV	Copepod (holoplankton)	3.80		0.40–0.60	2016–2019
*Tintinnopsis* spp.	Ciliate (planktonic)	10.20		0.09–0.11	1991–1993, 1995, 1998–2006, 2008, 2010–2018
*Tintinnopsis brandti*	Ciliate (planktonic)	0.90		0.14–0.18	2012–2013, 2015, 2018
*Tintinnopsis tubulosa*	Ciliate (planktonic)	0.30		0.10	2012
*Tintinnopsis fimbriata*	Ciliate (planktonic)	0.30		0.05–0.07	2013, 2018
*Tintinnopsis lobiancoi*	Ciliate (planktonic)	4.20		0.04–0.06	2013–2019
*Tintinnopsis beroidea*	Ciliate (planktonic)	2.40		0.05–0.07	2015–2019
*Vorticella* sp.	Ciliate (sessile)	7.00		0.15	1992-1994-1995, 2008, 2010–2019

## APPENDIX

Observed taxa, ecological group, relative appearance in samples, % of total calculated biomass, length (mm), and year of occurrence in the samples for all the taxa in the samples during the study period 1966–2019. Taxa included in the statistical analyses are indicated in bold.

## References

[1] Meier H.E.M., Kniebusch M., Dieterich C., Gröger M., Zorita E., Elmgren R., Myrberg K., Ahola M.P. (2022). Climate change in the Baltic Sea region: a summary. Earth System Dynamics.

[2] Beaugrand G. (2015). Synchronous marine pelagic regime shifts in the Northern Hemisphere. Philos. Trans. R. Soc., B.

[3] Reusch T. B. H. and others (2018). The Baltic Sea as a time machine for the future coastal ocean. Sci. Adv..

[4] Kessler A., Goris N., Lauvset K. (2022). Observation-based Sea surface temperature trends in Atlantic large marine ecosystems. Prog. Oceanogr..

[5] Leppäranta M., Myrberg K. (2009).

[6] Bergström S., Carlsson B. (1994). River runoff to the Baltic Sea 1950-1990. Ambio.

[7] Dippner J., Möller C., Hänninen J. (2012). Regime shifts in North Sea and Baltic Sea: a comparison. J. Mar. Syst..

[8] Hänninen J., Vuorinen I., Rajasilta M., Reid P.C. (2015). Response of the baltic and north seas to river runoff from the baltic watershed. — physical and biological changes. Prog. Oceanogr..

[9] Kändler R. (1949). Die Häuﬁgkeit pelagischer Fisheier in der Ostsee als Masstab für die Zu- und abnahme der Fischbestände. Kiel. Meeresforsch..

[10] Hela I. (1951). On the occurrence of the jellyﬁsh *Aurelia aurita* L. on the south coast of Finland. Arch. Soc. Zool. Bot. Fenn.

[11] Purasjoki K. (1958). Beobactungen über die Einwirkung gesteigerten Salzgehalts auf das Auftreten einiger marinen Zooplanktonarten ausserhalb Helsinki. Arch. Soc. Zool. Bot. Fenn.

[12] Segerstråle S.G. (1969). Biological ﬂuctuations in the Baltic Sea. Prog. Oceanogr..

[13] Vuorinen I., Hänninen J., Viitasalo M., Helminen U., Kuosa H. (1998). Proportion of copepod biomass declines together with decreasing salinities in the Baltic Sea. ICES J. Mar. Sci..

[14] Möllmann C., Kornilovs G., Sidrevics L. (2000). Long-term dynamics of main mesozooplankton species in the central Baltic Sea. J. Plankton Res..

[15] Hänninen J., Vuorinen I., Hjelt P. (2000). Climatic factors in the Atlantic control the oceanographic and ecological changes in the Baltic Sea. Limnol. Oceanogr..

[16] Hansson S., Larsson U., Johansson S. (1990). Selective predation by herring and mysids, and zooplankton community structure in a Baltic Sea coastal area. J. Plankton Res..

[17] Österblom H., Hansson S., Larsson U., Hjerne O., Wulff F., Elmgren R., Folke C. (2007). Human-induced trophic cascades and ecological regime shifts in the Baltic Sea. Ecosystems.

[18] Tomczak M.T. (2022). Reference state, structure, regime shifts, and regulatory drivers in a coastal sea over the last century: the Central Baltic Sea case. Limnol. Oceanogr..

[19] Hernroth L. (1985). Recommendations on methods for marine biological studies in the Baltic Sea. Mesozooplankton assessment 10.

[20] Levander K.M. (1900). Über das Herbst- und Winterplankton im Finnischen Meerbusen und in der Ålands-See. Acta Soc. Fauna Flora Fenn..

[21] Leegaard C. (1920). Microplankton from the Finnish waters during the month of May 1912. Acta Soc. Fauna Flora Fenn..

[22] Hurrell J.W. (1995). Decadal trends in the North Atlantic oscillation: regional temperatures and precipitation. Science.

[23] Hänninen J. (2022). The Baltic Sea ecosystem regulation mechanism. Environ. Anal. Eco. Stud..

[24] Alheit J., Möllmann C., Dutz J., Kornilovs G., Loewe P., Morholz V., Wasmund N. (2005). Synchronous ecological regime shifts in the central Baltic and the North Sea in the late 1980s. ICES J. Mar. Sci..

[25] Beamish R.J., Noakes D.J., Mcfarlane G.A., Pinnix W., Sweeting R., King J. (2000). Trends in coho marine survival in relation to the regime concept. Fish. Oceanogr..

[26] Jiao Y. (2008). Regime shift in marine ecosystems and implications for fisheries management, a review. Rev. Fish Biol. Fish..

[27] Möllmann C., Diekmann R., Müller-Carulis B., Kornilovs G., Plikshs M., Axe P. (2009). Reorganization of a large marine ecosystem due to atmospheric and anthropogenic pressure: a discontinuous regime shift in the Central Baltic Sea. Global Change Biol..

[28] Conley D.J., Humborg C., Rahm L., Savchuk O.P., Wulff F. (2002). Hypoxia in the Baltic Sea and basin-scale changes in phosphorus biogeochemistry. Environ. Sci. Technol..

[29] Jansson A., Klais-Peets R., Grinienė E., Rubene G., Semenova A., Lewandowska A., Engström-Öst J. (2020). Functional shifts in estuarine zooplankton in response to climate variability. Ecol. Evol..

[30] Nehring D., Matthäus W. (1991). Current trends in hydrographic and chemical parameters and eutrophication in the Baltic Sea. Int. Rev. Gesamten Hydrobiol. Hydrogr..

[31] Wasmund N. (2001). Trophic status of the south-eastern Baltic Sea: a comparison of coastal and open areas. Estuar. Coast Shelf Sci..

[32] Leppäkoski E., Helminen H., Hänninen J., Tallqvist M. (1999). Aquatic biodiversity under anthropogenic stress: an insight from the Archipelago Sea (SW Finland). Biodivers. Conserv..

[33] Väänänen K., Lappalainen M., Högmander J. (2020). The Unique Archipelago Sea.

[34] Voipio A. (1981).

[35] Hänninen J., Vuorinen I., Helminen H., Kirkkala T., Lehtilä K. (2000). Trends and gradients in nutrient concentrations and loading in the Archipelago Sea, northern baltic, in 1970–1997. Estuar. Coast Shelf Sci..

[36] HELCOM (1988). Guidelines for the baltic monitoring programme for the third stage. Part D. Biological determinands. *Balt*. Sea Environ. Proc..

[37] Mäkinen K., Vuorinen I., Hänninen J. (2017). Climate-induced hydrography change favors small-bodied zooplankton in a coastal ecosystem. Hydrobiologia.

[38] Rajasilta M., Vuorinen I. (2008). Suomen murtovesialueen eläinplankton –määritysopas [Zooplankton analysis guide for the Finnish brackish water area]. SEILI Archipel. Res. Inst. Publ..

[39] Burris J.E. (1980). Vertical migration of zooplankton in the Gulf of Finland. Am. Midl. Nat..

[40] Carter J.C.H., Goudie K.A. (1986). Diel vertical migrations and horizontal distributions of *Limnocalanus macrurus* and *Senecella* calanoides (copepoda, calanoida) in Lakes of Southern Ontario in relation to planktivorous fish. Can. J. Fish. Aquat. Sci..

[41] Antsulevich A. (2020). Monitoring of alien species Cercopagis pengoi in the Hanko area in1998-2005, Centre for Economic Development, Transport, and the Environment for. Uusimaa.

[42] SAS Institute Inc (2013).

[43] McCulloch C.E., Searle S.R., Neuhaus J.M. (2008).

[44] Breslow N.E., Clayton D.G. (1993). Approximate inference in generalized linear mixed models. J. Am. Stat. Assoc..

[45] SAS Institute Inc (2020).

[46] Schinke H., Matthäus W. (1998). On the causes of major Baltic inflows - an analysis of long time series. Continent. Shelf Res..

[47] Matthäus W., Schinke H. (1994). Mean atmospheric circulation patterns associated with major Baltic inflows. *Dtsch. Hydrogr*. Zeitschrift.

[48] Vuorinen I., Hänninen J., Kornilovs G. (2003). Transfer-function modelling between environmental variation and mesozooplankton in the Baltic Sea. Prog. Oceanogr..

[49] Hänninen J., Vuorinen I., Kornilovs G. (2003). Atlantic climatic factors control decadal dynamics of a Baltic Sea Copepod, Temora longicornis. Ecography.

[50] Gillet N.P., Graf H.F., Osborn T.J. (2003). Climate change and the North Atlantic oscillation. Geophys. Monogr..

[51] Hansson S., Dippner J.W., Larsson U. (2010). Climate effects on zooplankton biomasses in a coastal Baltic Sea area. Boreal Environ. Res..

[52] Rajasilta M., Hänninen J., Laaksonen L., Laine P., Suomela J.-P., Vuorinen I., Mäkinen K. (2019). Influence of environmental conditions, population density, and prey type on the lipid content in Baltic herring (Clupea harengus membras) from the northern Baltic Sea. Can. J. Fish. Aquat. Sci..

[53] Ojaveer H., Simm M., Lankov A. (2004). Population dynamics and ecological impact of the non-indigenous *Cercopagis pengoi* in the Gulf of Riga (Baltic Sea). Hydrobiologia.

[54] Dippner J., Kornilovs G., Sidrevics L. (2000). Long-term variability of mesozooplankton in the central Baltic Sea. J. Mar. Syst..

[55] Strecker A.L., Cobb T.P., Vinebrooke R.D. (2004). Effects of experimental greenhouse warming on phytoplankton and zooplankton communities in fishless alpine ponds. Limnol. Oceanogr..

[56] Hänninen J. (1999).

[57] Viitasalo M., Bonsdorff E. (2022). Global climate change and the Baltic Sea ecosystem: direct and indirect effects on species, communities and ecosystem functioning. Earth Syst. Dynam..

[58] Viitasalo M., Vuorinen I., Saesmaa S. (1995). Mesozooplankton dynamics in the northern Baltic Sea: implications of variations in hydrography and climate. J. Plankton Res..

[59] Flinkman J., Aro E., Vuorinen I., Viitasalo M. (1998). Changes in northern Baltic zooplankton and herring nutrition from 1980s to 1990s: top-down and bottom-up processes at work. Mar. Ecol. Prog. Ser..

[60] Keen E. (2013). A practical designer's guide to mesozooplankton nets. https://acsweb.ucsd.edu/%7Eekeen/resources/Choosing-a-Net.pdf.

[61] Jaagus J., Sepp M., Tamm T., Järvet A., Mõisja K. (2017). Trends and regime shifts in climatic conditions and river runoff in Estonia during 1951–2015. Earth Syst. Dynam..

[62] Rudstam R.G., Hansson S., Johansson S., Larsson U. (1992). Dynamics of planktivory in a coastal area of the northern Baltic Sea. Mar. Ecol. Prog. Ser..

[63] Möllmann C., Köster F.W. (1999). Food consumption by clupeids in the Central Baltic: evidence for top-down control?. ICES J. Mar. Sci..

[64] Vuorinen I., Ranta E. (1987). Dynamics of marine meso-zooplankton at Seili, northern Baltic Sea, in 1967-1975. Ophelia.

[65] Bradley D.C., Ormerod S.J. (2001). Community persistence among stream invertebrates tracks the North Atlantic Oscillation. J. Anim. Ecol..

[66] Mäkinen K. (2019).

[67] Andersson A. and others (2015). Projected future climate change and Baltic Sea ecosystem management. Ambio.

